# A ubiquinone precursor analogue does not clearly increase the growth rate of *Caenorhabditis inopinata*

**DOI:** 10.17912/micropub.biology.001235

**Published:** 2024-12-05

**Authors:** Gavin C. Woodruff, Kimberly A. Moser

**Affiliations:** 1 School of Biological Sciences, University of Oklahoma, Norman, Oklahoma, United States

## Abstract

The evolution of developmental rates may drive morphological change. *Caenorhabditis inopinata* develops nearly twice as slowly as *Caenorhabditis elegans* . *clk-1* encodes a hydroxylase required for synthesizing ubiquinone, and mutant *clk-1* slow growth phenotypes can be rescued by supplying animals with a ubiquinone precursor analogue, 2,4-dihydroxybenzoate. RNA-seq data showing low *clk-1* expression raised the possibility that *C. inopinata* grows slowly because of reduced ubiquinone biosynthesis. *C. inopinata* did not reveal a clear reduction in the age of maturation when reared on 2,4-dihydroxybenzoate. Further scrutiny of RNA-seq results revealed multiple ubiquinone metabolism genes have low expression in *C. inopinata* . Divergent *clk-1* expression alone may not be a major driver of the evolution of slow development in this species.

**Figure 1. A ubiquinone precursor analogue does not clearly increase the growth rate of C. inopinata:**
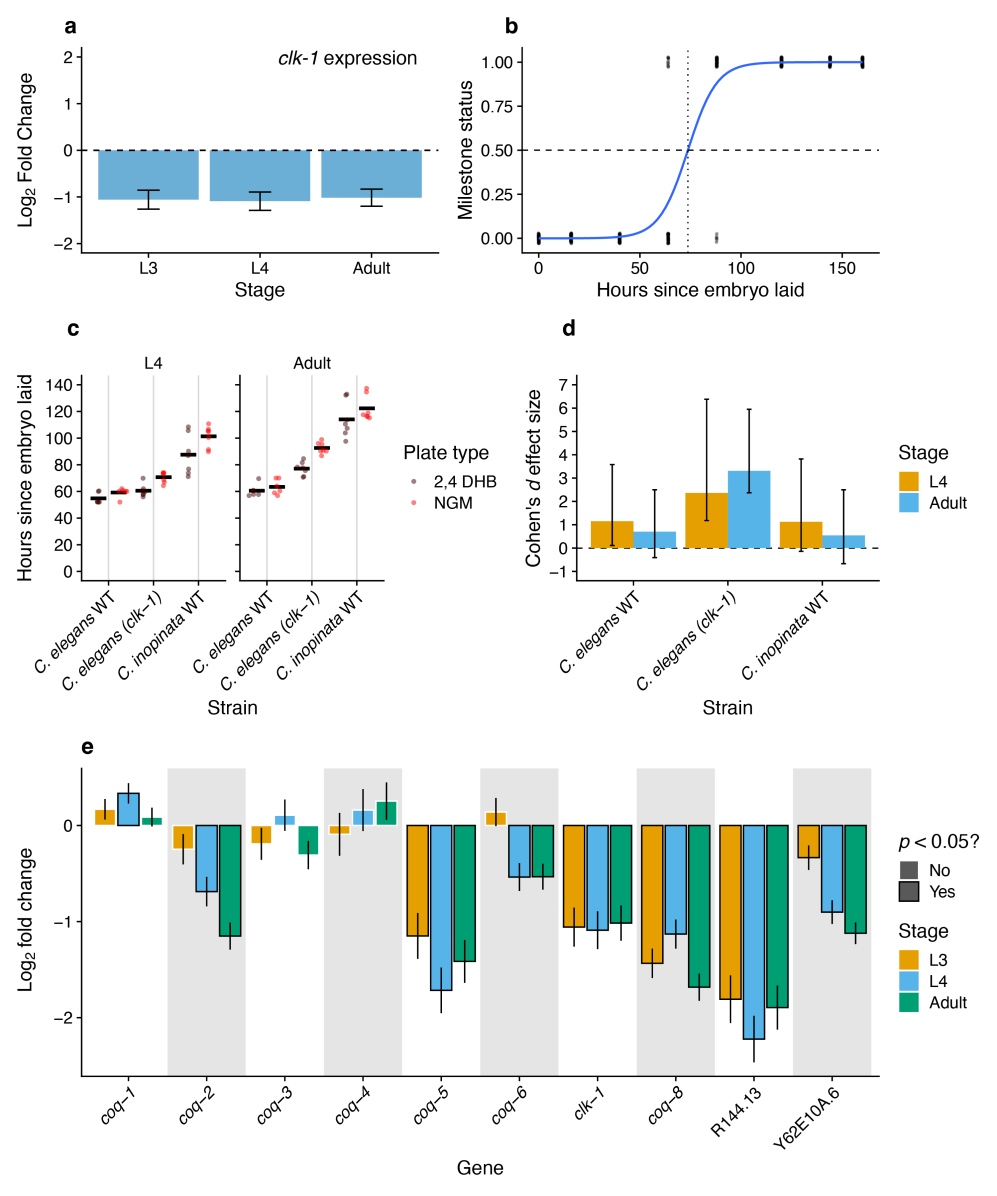
( **a** ) *clk-1* exhibits a one-fold decrease in expression across three developmental stages in *C. inopinata* compared to *C. elegans* . Data from (Woodruff et al. 2024). ( **b** ) Estimates of developmental timing were made with logistic models. For a given plate, the number of worms at a given developmental stage (in this example, the L4 stage) were counted at least once per day until all animals reached adulthood. The number of worms younger than the L4 stage (Milestone status = 0) or at the L4 stage and older (Milestone status = 1) were plotted over time. A logistic model was fit to this data (solid blue line), and the time at which 50% of the animals were predicted to have passed the milestone was determined (dotted and dashed lines). These estimates of median time to the developmental milestone were then plotted (c) and analyzed. ( **c** ) The timing of developmental events on DHB. The y-axis represents the median time to the developmental milestone as inferred in (b). Each point represents a plate. Sina plots are strip charts with points taking the contours of a violin plot; black bars represent means. *C. elegans* WT, PD1074; *C. elegans (clk-1)* , MQ130; *C. inopinata* WT, NKZ35. The different facets represent the times to the L4 and adult developmental milestones. ( **d** ) Cohen's *d* effect sizes of DHB compared to NGM plates without the compound (using the data shown in (c)). Positive values represent faster developmental rates on DHB. Error bars represent 95% confidence intervals generated with one thousand bootstrap replicates of the data. ( **e** ) Many ubiquinone synthesis pathway genes are downregulated in *C. inopinata* . Plotted are the log _2_ fold changes in RNA expression of ubiquinone synthesis pathway genes at each developmental stage between *C. elegans* and *C. inopinata* . Data from (Woodruff et al. 2024 *G3* ). Genes included here are homologs of yeast genes previously reported to contribute to the synthesis of ubiquinone from synthetic analogues (Xie et al. 2012). R144.13 is a homolog of the yeast gene *COQ10* (by best reciprocal *blastp* hit). Y62E10A.6 is a homolog of the yeast gene *ARH1* (by best reciprocal *blastp* hit), which may participate in ubiquinone synthesis (Pierrel et al. 2010). There are no clear single-copy orthologs of the yeast gene *COQ9* in *C. elegans* . *YAH1* (implicated in ubiquinone synthesis in yeast (Pierrel et al. 2010)) has a single-copy ortholog in *C. elegans* (Y73F8A.27), but this gene has no clear best-reciprocal blast hit in *C. inopinata* . *clk-1* is the nematode homolog of the yeast gene *COQ7* . All *p* -values were corrected for multiple tests, and error bars represent 95% confidence intervals (see (Woodruff et al. 2024) for details regarding RNA-seq data and analysis).

## Description

Within evolutionary developmental biology, the evolution of developmental timing (or heterochrony) has been proposed to be a major driver of morphological change (Gould, 1977) . Heterochrony is most frequently discussed in terms of the *relative* timing of developmental events (Gould, 1977; McKinney & McNamara, 1991) . Changes of the rate, onset, and offset of growth of one structure relative to another (within an organism) hold the potential to explain myriad diverse morphological traits (Alberch et al., 1979; McNamara, 2012; McKinney & McNamara, 1991) . Yet, *global* changes in developmental rates (across all tissues) also occur and can have profound impacts on life histories, as generation time frequently covaries with body size, fecundity, and lifespan. *Caenorhabditis inopinata* , the closest known relative of *C. elegans* (Kanzaki et al., 2018) , reveals a global decrease in developmental rate compared to its sister species, with a maturation time that is nearly twice as long (Woodruff et al., 2019) . As *C. elegans* is a widely-used genetic model system, comparative studies using *C. inopinata* hold the potential to uncover the genetic bases of developmental rate evolution. Moreover, the vast knowledge base associated with the *C. elegans* system affords the ability to generate testable hypotheses regarding these potential genic drivers.

The *C. elegans* gene *clk-1* encodes a hydroxylase required for the synthesis of ubiquinone, a component of the election transport chain (Branicky et al., 2000; Ewbank et al., 1997; Felkai et al., 1999) . This gene is highly pleiotropic, with loss-of-function mutations promoting delayed hatching and maturation; longer cell cycles; lower fecundity; longer defecation cycles; slower pharyngeal pumping rates; and longer lifespans (Wong et al., 1995) . Mutations in *clk-1* can more than double the maturation time compared to wild-type animals and lead to a 70% reduction in brood size (Wong et al., 1995) . Moreover, some alleles of *clk-1* not only promote delayed development, they also reveal higher variance in developmental rates (Wong et al., 1995) . These mutant phenotypes bear striking resemblances to the divergent *C. inopinata* : *C. inopinata* takes nearly twice as long to develop as *C. elegans* ; has more variability in developmental rates (Woodruff et al., 2018, 2019) ; and harbors brood sizes that are an order of magnitude smaller (Woodruff et al., 2019) . Additionally, as *C. inopinata* is likely to have a similar number of cells as *C. elegans* (Woodruff et al., 2018) , this implies its cell cycles are of longer duration. Taken together, these observations suggest the possibility that the slow growth of *C. inopinata* may be tied to reduced *clk-1* activity. Indeed, a recent comparative transcriptomics study (Woodruff et al., 2024) revealed a one-fold decrease in *clk-1* gene expression in *C. inopinata* compared to *C. elegans* at all developmental stages ( Figure 1A ; Wald test Holm-corrected p < 0.0001 for all comparisons; Woodruff et al., 2024). Thus, *clk-1* transcript abundance is lower in *C. inopinata* compared to *C. elegans* , consistent with its slower growth rate.

However, it was unclear to what extent decreased *clk-1* transcript abundance influences the global rate of development in *C. inopinata* . In *C. elegans* , many *clk-1* mutant phenotypes can be rescued via the addition of the alternative ubiquinone precursor 2,4-dihydroxybenzoate (2,4-DHB) (Liu et al., 2017) . If the biochemistry of ubiquinone synthesis (and the function of *clk-1* ) is conserved among species, and if reduced *clk-1* activity contributes to reduced growth rates in *C. inopinata* , then the addition of 2,4-DHB would be predicted to increase the growth rate of *C. inopinata* by restoring electron transport chain function. To test this, we reared *C. inopinata* on 2,4-DHB. 2,4-DHB did not promote a significant change in the maturation time of wild-type *C. elegans* ( Figure 1C ; NGM average maturation time = 63 hours; 2,4-DHB average maturation time = 61 hours; Wilcoxon rank-sum test adjusted *p* = 0.485; Cohen's *d* effect size = 0.705; N _plates_ = 6 per group; N _embryos per plate_ = 53-131), whereas 2,4-DHB did reduce the maturation time of *C. elegans clk-1 ( qm30 )* mutants ( Figure 1C ; NGM average maturation time = 93 hours; 2,4-DHB average maturation time = 77 hours; Wilcoxon rank-sum test adjusted *p* = 0.001; Cohen's *d* effect size = 3.31; N _plates_ =7 per group; N _embryos per plate_ = 54-136). And, 2,4-DHB did not promote a significant change in the maturation time of *C. inopinata* , although 2,4-DHB-treated animals matured slightly more quickly ( Figure 1C ; NGM average maturation time = 122 hours; 2,4-DHB average maturation time = 114 hours; Wilcoxon rank-sum test adjusted *p* = 0.078; Cohen's *d* effect size = 0.544; N _plates_ =7 per group; N _embryos per plate_ = 47-152).

While *clk-1* exhibits lower expression in *C. inopinata* ( Figure 1A ; Woodruff et al., 2024), supplementing *C. inopinata* with a ubiquinone precursor analogue does not clearly speed up its rate of growth ( Figure 1C -D). 2,4-DHB is capable of rescuing mutant *clk-1* phenotypes because other enzymes in the ubiquinone synthesis pathway can metabolize this substrate to ubiquinone independently of CLK-1 (Liu et al., 2017; Xie et al., 2012) . Many biochemical steps and enzymes (at least six) constitute this alternative pathway (Xie et al., 2012) . One possibility why 2,4-DHB does not increase the growth rate of *C. inopinata* is that the activity of these other enzymes is also low. Indeed, a broader picture of the transcriptional activity of ubiquinone synthesis genes reveals that two of these enzymes are also downregulated in *C. inopinata* at all developmental stages ( Figure 1E ; *coq-5* and the homolog of the yeast gene *ARH1* ( Y62E10A.6 ) (Pierrel et al., 2010; Woodruff et al., 2024) ). Two other enzymes reveal significant differential expression at the L4 and adult stages ( Figure 1 e; *coq-2* and *coq-6* ). *coq-3* and *coq-4* reveal no significant differences in expression between *C. elegans* and *C. inopinata* at any developmental stage ( Figure 1E ; Woodruff et al., 2024). *coq-1 , coq-8 ,* and R144.13 (homolog of yeast *COQ10* ) are not clearly implicated in this alternative 2,4-DHB metabolic pathway, but they are implicated in other ubiquinone synthesis pathways (Xie et al., 2012) . *coq-1* expression is elevated in *C. inopinata* in the L4 stage, but is not differentially expressed at other stages ( Figure 1E ; Woodruff et al., 2024). *coq-8* and R144.13 (homolog of yeast *COQ10* ) exhibit lower expression in *C. inopinata* at all developmental stages ( Figure 1E ; Woodruff et al., 2024). Thus, one plausible reason 2,4-DHB does not cause faster development in *C. inopinata* is this alternative metabolic pathway is also downregulated in this species.

Yet, this is not the only possible biochemical explanation for our results. Crucially, substrate concentrations and protein-level enzyme activities have not been measured in our laboratory populations. It is possible that despite their lower expression, these enzymes have increased protein-level metabolic activity (i.e., these enzymes could more quickly catalyze their respective reactions). This kind of offset at the protein-level would also promote a lack of 2,4-DHB impact on developmental rate—in this case, low *clk-1* expression would not cause comparatively lower *clk-1* activity as the protein it encodes would be a faster catalyst. Along the same lines, if low CLK-1 activity *were* the sole cause of low developmental rates in *C. inopinata* , then we would expect elevated concentrations of its substrate (6-demethoxyubiquinone; (Liu et al., 2017; Xie et al., 2012) ) in *C. inopinata* . Lower (or comparable) concentrations of 6-demethoxyubiquinone in *C. inopinata* would have suggested other enzymes are capable of metabolizing this substrate to ubiquinone. Another possibility, consistent with our RNA-seq data, is that multiple components of this metabolic pathway have reduced activity, and that the addition of 2,4-DHB is insufficient to circumvent low enzymatic activity across multiple edges of the ubiquinone synthesis network. Moreover, we also have not measured product concentrations; it is possible that ubiquinone concentrations are comparable among species. In this case, low transcript abundances of ubiquinone enzyme transcripts may have been offset by compensatory changes elsewhere in *C. inopinata* . Regardless, it is likely that reduced *clk-1* transcriptional activity alone cannot entirely explain the elongated developmental period of this species.

Beyond this, *C. inopinata* evolved to thrive in fresh figs from ancestors that lived in plant detritus (Kanzaki et al., 2018; Kiontke et al., 2011) , and it is likely that widespread changes in its metabolism and development have occurred as a consequence. As *Caenorhabditis* nematodes are bacterivores, *C. elegans* and *C. inopinata* are likely to consume divergent bacterial resources (Dirksen et al., 2016; Kanzaki et al., 2018; Woodruff & Phillips, 2018) , which is expected to co-occur with changes in metabolic pathways (MacNeil et al., 2013; Watson et al., 2014) . *C. inopinata* also thrives in an enclosed-fig environment (Kanzaki et al., 2018; Woodruff & Phillips, 2018) that is likely to be oxygen-poor as well as light-depleted— these drastic environmental changes may have impacts on development and metabolism (Hussey et al., 2018; Migliori et al., 2011) . Additionally, *C. inopinata* disperses on fig wasps (Woodruff & Phillips, 2018) . As the *C. inopinata* dauer decision is likely to be timed to fig ripening and wasp emergence (Hammerschmith et al., 2022; Woodruff & Phillips, 2018) , this could promote differences in the regulation of developmental timing (or metabolism). *C. inopinata* is also much larger than *C. elegans* (Kanzaki et al., 2018; Woodruff et al., 2018) , and there may well be connections between the genetic regulatory networks underlying body size and growth rate divergence (Calder, 1984; Peters, 1983) . Additionally, decades of genetic studies have revealed key developmental pathways that regulate the relative timing of developmental events as well as the molting cycle in *C. elegans* (Lažetić & Fay, 2017; Moss, 2007), and the extent to which these pathways have been modified in *C. inopinata* remains an open question. Regardless, a ubiquinone precursor analogue does not cause *C. inopinata* to grow as quickly as *C. elegans* , and the causes of developmental rate evolution are likely numerous.

## Methods

*Strains and media*

The strains *C. elegans* PD1074 (a re-sequenced inbred wild-type strain) (Yoshimura et al., 2019) ; *C. elegans* *clk-1 ( qm30 )* MQ130 (Lakowski & Hekimi, 1996; Wong et al., 1995) ; and *C. inopinata* NKZ35 (inbred reference strain) (Kanzaki et al., 2018) were used for this study. Animals were reared on Nematode Growth Media plates (supplemented with 3% agar to prevent burrowing) seeded with *E. coli* OP50 . To test the impact of a ubiquinone precursor analogue on developmental rates, 2,4-dihydroxybenzoate was added to the media at a final concentration of 1 mM before pouring plates (Liu et al. 2017) .

*Developmental timing estimates*

Animals were synchronized by time-limited egg-laying. 50 *C. elegans* hermaphrodites (or 100 *C. inopinata* gravid females) per plate were plated and then removed after three hours. The number of embryos laid was counted, and the plates were periodically scored (at least once per day) for distributions of animals at respective developmental stages. Animals were reared at 20°C. For every observation, the number of animals at respective developmental stages (L1-L3 (larval), L4, adult) were counted until the number of adults did not increase for two days (and the production of the next generation obscured the inference of developmental rates). Median times to the L4 and adult developmental milestones were inferred as in (Woodruff et al., 2019) . Briefly, for each plate and each day, animals were coded by L4 and adult milestone status (0, before milestone; 1, at or after milestone). These values were plotted over time, and logistic models were fit using the *glm()* function (with option family="binomial") in the R statistical programming language. Estimates of the median time to the L4 and adult milestones were extracted from these models for each plate ( Figure 1B ). These plate medians were then compared among experimental groups to infer differences in developmental rates (Figures 1C-D).

*Transcriptomics*

RNA-seq methods, data, and analyses were reported in (Woodruff et al., 2024) .

*Statistical analyses*

Statistics were performed using the R programming language. Pairwise Wilcoxon rank-sum tests and inferences of Cohen's d effect sizes among groups were performed in R with the *rstatix* package in R; *p* -values were corrected for multiple comparisons (Benjamini & Hochberg, 1995) . All data and code affiliated with this work have been deposited in Github ( https://github.com/gcwoodruff/clk-1_micropub ). Data are also available as extended data at *microPublication Biology* .

## Extended Data

Description: A zip file of the Github release "clk-1_micropub". This contains R code, developmental timing data, and transcript count data for the genes examined in this paper. Resource Type: Software. DOI: 10.22002/3wvmb-5c709
